# Mitochondrial dysfunction and its role in tissue-specific cellular stress

**DOI:** 10.15698/cst2018.07.147

**Published:** 2018-07-13

**Authors:** David Pacheu-Grau, Robert Rucktäschel, Markus Deckers

**Affiliations:** 1 Department of Cellular Biochemistry, University Medical Center Göttingen, Germany.

**Keywords:** mitochondrial dysfunction, cellular stress, mitochondrial pathology, therapy

## Abstract

Mitochondrial bioenergetics require the coordination of two different and independent genomes. Mutations in either genome will affect mitochondrial functionality and produce different sources of cellular stress. Depending on the kind of defect and stress, different tissues and organs will be affected, leading to diverse pathological conditions. There is no curative therapy for mitochondrial diseases, nevertheless, there are strategies described that fight the various stress forms caused by the malfunctioning organelles. Here, we will revise the main kinds of stress generated by mutations in mitochondrial genes and outline several ways of fighting this stress.

## MITOCHONDRIA AND CELL METABOLISM

Mitochondria play a pivotal role in eukaryotic metabolism. They catabolise redox equivalents, derived from nutrient uptake, and use them to provide the bulk of cellular energy in the form of ATP. The oxidative phosphorylation system (OXPHOS) is responsible for this energy production and it is composed of five multi-oligomeric complexes present in the inner mitochondrial membrane. Transfer of electrons through complexes I to IV reduce molecular oxygen to water. This process is coupled to proton pumping from the matrix to the intermembrane space (IMS), while the return of protons to the matrix through the F1Fo ATPase generates ATP 1. However, an inefficient flow of electrons through the respiratory chain complexes would partially reduce oxygen and produce reactive oxygen species (ROS) like superoxide and hydrogen peroxide. At low concentrations, these molecules act as second messengers and can activate gene transcription and trigger cellular responses, like cellular growth, production of cellular antioxidants or stimulation of mitochondrial biogenesis 2,3. However, once a certain threshold is exceeded, these molecules may incite oxidative damage in the form of mitochondrial DNA (mtDNA) alterations or lipid peroxidation, generating cellular stress that leads to aging or cell death.

In addition, mitochondria are involved in many other key cellular functions. Dissipation of the proton gradient by uncoupling proteins (UCPs) generates heat instead of energy and this plays an important role in exposure to cold or hibernation 4. Calcium (Ca^2+^) uptake inside mitochondria is mediated by the mitochondrial calcium uniporter (MCU). Although the complex has a low affinity for Ca^2+^, the transport takes place due to the high concentration of Ca^2+^ (>10 μM) present in micro domains located in the contact sites between endoplasmic reticulum (ER) and mitochondria 5. The mitochondrial Ca^2+^ uptake not only shapes the cytosolic Ca^2+^ dynamics, which is crucial for muscle contraction, exocytosis and gene transcription, but also modulates at least three dehydrogenases of the Krebs cycle, thus regulating energy metabolism. Finally, Ca^2+^ overload in mitochondria regulates apoptosis due to formation of the permeable transition pore (PTP) and release of cytochrome *c* from the IMS 6. Mitochondria are involved in the biogenesis and maturation of different cofactors, like heme, biotin or iron-sulfur (Fe/S) clusters. Despite the chemical simplicity of Fe/s clusters, their biosynthesis requires more than two dozen proteins in eukaryotes and takes place both in mitochondria and the cytosol 7. Alterations in these mechanisms are linked to severe neurodegenerative, metabolic or haematological diseases 8.

Since mitochondria take part in many different metabolic processes, mitochondrial malfunction can affect numerous aspects of the cell. As a consequence, various forms of cellular stress are generated, leading to a large variety of pathological conditions. Here, we review different forms of cellular stress caused by mitochondrial malfunction and the strategies used to fight this stress.

## MITOCHONDRIAL DEFECTS

Mitochondria have retained their own genome, the mtDNA. This small, circular, double-stranded DNA is located in the mitochondrial matrix in all cell types, and can be found with copy numbers that range from several to thousands of copies. In human, the mtDNA encodes 13 polypeptides of the respiratory chain, as well as for part of the translation machinery, required for the synthesis of these polypeptides within mitochondria: two ribosomal RNAs (mt-rRNAs) and 22 transfer RNAs (mt-tRNAs) 9. The remaining mitochondrial proteins (approx. 99%) are encoded in the nucleus, synthesized on the cytosolic ribosomes and imported into mitochondria. Therefore, we will distinguish between mitochondrial malfunction caused by mutations in the mtDNA and those caused by mutations of nuclear genes encoding mitochondrial proteins.

### Alterations in the mtDNA

Some features of mtDNA make it especially sensitive to oxidative damage and mutation. Firstly, mtDNA has no introns, so every single nucleotide carries information essential for protein coding; mtDNA is naked, there are no histone proteins protecting it from damage; and although DNA repair systems do exist in mitochondria, their mechanisms and extent are poorly understood 10, therefore mutations usually remain and are transmitted to the next generation until they are removed by selection^11^. Moreover, the proximity to the respiratory chain, a ROS producing source, increases the risk of potential damage. For all these reasons, the mutational rate of the mitochondrial genome is much higher than that of the nuclear genome 12.

**Figure 1:**
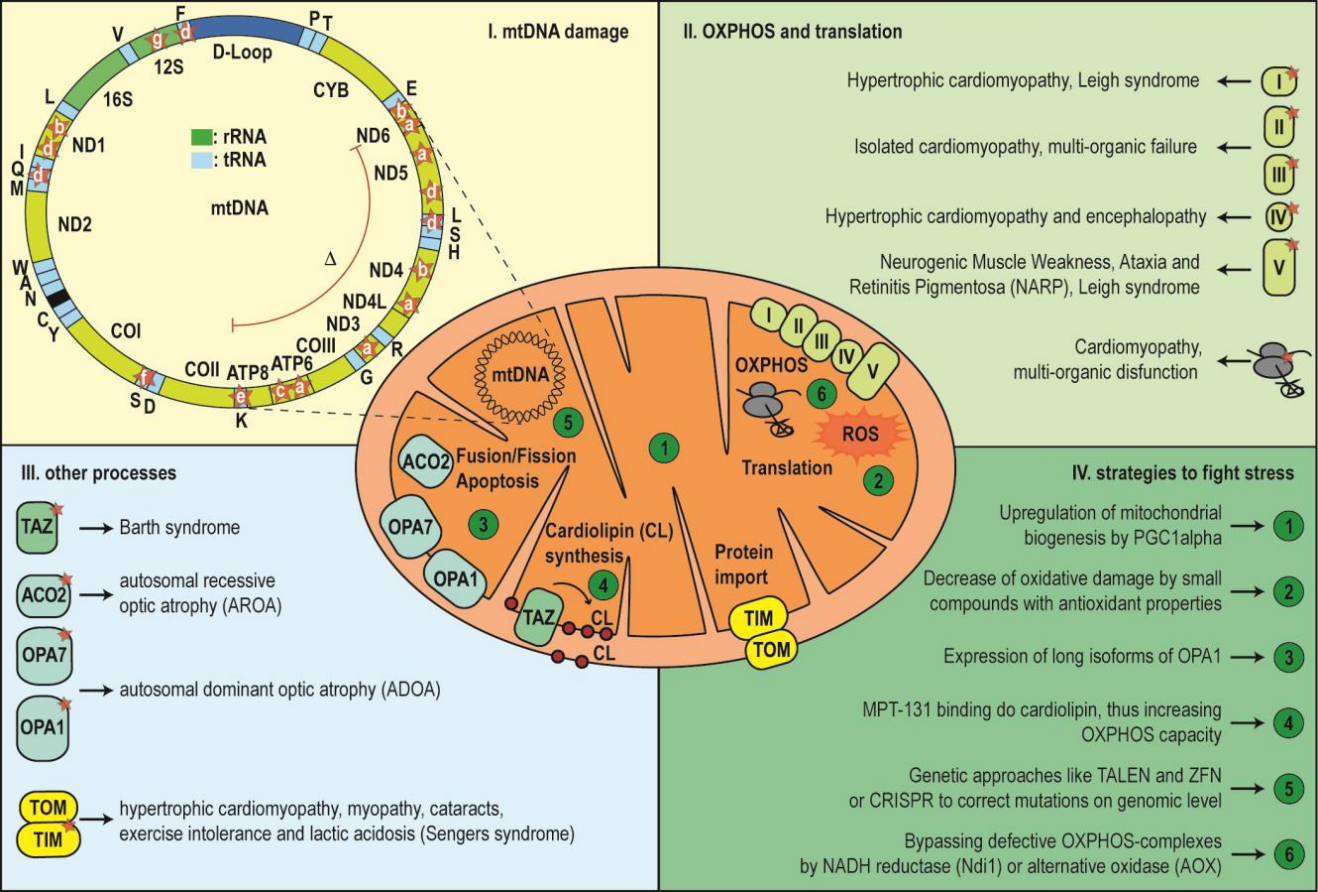
FIGURE 1: Mitochondrial dysfunctions are related to mutations in mtDNA and defects in nuclear encoded mitochondrial proteins. **(I)** Overview of mutations within the mtDNA. **(II)** The majority of mitochondrial defects based on a malfuntion of OXPHOS complexes and mitochondrial translation. **(III)** Defects in other processes, like mitochondrial fusion and fission or lipid homeostasis, leads to different mitochondrial diseaes. **(IV)** Different strategies to figth the diverse forms of mitochondrial stress. (**a:** Leigh Syndrome **LS**; **b:** Leber Hereditary Optic Neuropathy **LHON**; **c:** Neurogenic Muscle Weakness, Ataxia and Retinitis Pigmentosa **NARP**; **d:** Mitochondrial Encephalomyopathy, Lactid Acidosis and Stroke-like Episodes **MELAS**; **e:** Myoclonic Epilepsy and Ragged Red Fiber Disease **MERRF**; **f:** Sensorineural Hearing Loss **SNHL**; **g:** mitochondrial non-syndromic Hearing Loss; **(:** Kearns Sayre Syndrome **KSS**).

Pathological changes in the mtDNA can appear as point mutations in protein coding sequences, mt-tRNAs or even mt-rRNAs. In addition, major rearrangements of mtDNA, like deletions or insertions/duplications, are a cause of disease. Due to the fact that every cell contains a variable number of mtDNA molecules, mutations can be present in homoplasmy (all copies share the same mtDNA genotype) or heteroplasmy (only a population of DNA is mutated). The level of heteroplasmy of a mutation is a critical determinant of the cellular stress of a certain tissue or organ and has a major role in the disease phenotype. Finally, a reduction of mtDNA copies (depletion syndrome) can also hamper energy production and generate cellular stress (see **Table 1**, **Figure 1**) 12.

### Alterations in the mitochondrial proteins encoded in the nucleus

Due to the diverse cellular roles that mitochondria fulfill, there are many mitochondrial processes that cause a pathology when disturbed. In the last years, massive sequencing approaches have significantly increased the number of known mutations implicated in mitochondrial diseases. Examples of this are defects in: factors involved in the biogenesis or integrity of respiratory chain complexes, those that regulate mtDNA maintenance, proteins required for transcription of mt-mRNA elements involved in translation of mtDNA encoded proteins, regulators of lipid metabolism, factors involved in cellular signalling and even enzymes of the Krebs cycle (see **Table 2**).

## CELLULAR EFFECTS ON DIFFERENT TISSUES

Typically, mitochondrial disorders have been divided between those presenting with multiple symptoms, usually known as syndromes, and those characterized by tissue specific phenotypes. It remains to be addressed, which factors determine the tissue-specificity of mitochondrial diseases. However, to better address the different kinds of stress caused by mitochondrial distress, we will describe them classified by tissues/organs and give some examples of alterations that cause these problems.

### Sensory organs 

Hearing loss is one of the most prevalent sensory disorders 61. Genetic factors are thought to account for more than half of congenital and childhood-onset hearing loss, including mutations in mtDNA 62 and mitochondrial nuclear genes like the heme A biogenesis factor COX10 63,64 or the AAA protease responsible for complex III assembly BSC1L 65,66.

Mutations in the 12S mt-rRNA (m.1555A>G and m.1494 C>T) have been associated with aminoglycoside-induced ototoxicity and mitochondrial non-syndromic hearing loss. Studies using mitochondrial cybrids derived from Hela cells and lymphoblasts have shown that these mutations affect the integrity and fidelity of the mitochondrial ribosome, therefore causing decreased mitochondrial translation, either in the presence or the absence of aminoglycosides, and resulting in a cell growth defect 67,68.

However, a study using osteosarcoma 143B derived cybrids showed no effect on mitochondrial translation after aminoglycoside treatment 69.

This discrepancy, the phenotypic differences between asymptomatic relatives and patients all harbouring the same mutational load and the fact that only in some cases the defect arose upon antibiotic treatment, raised the search for modifying factors of aminoglycoside induced ototoxicity within the nuclear genetic background 70. Indeed, no negative effect was observed after aminoglycoside treatment in primate cells from the Cercophiteciade family where the m.1494 C>T was fixed as the wild-type allele and cells carried a compensating mutation in mitochondrial ribosomal protein S12 (MRPS12) 71, whereas primate cells from orangutan carrying the m.1555A>G mutation and no MRP mutation showed a drastic effect after antibiotic treatment 72. In addition, this biochemical effect has been linked to stress signalling. Cybrids carrying the m.1555A>G mutation showed hypermethylation of the mitochondrial ribosome, disturbed mitochondrial translation and assembly of the respiratory chain, resulting in increased production of ROS. Enhanced superoxide levels are sensed by AMPK, which signals further to E2F1, activating pro-apoptotic signalling in the cell. This induction seems to be tissue-specific, happens mainly in the inner ear and may explain the specific hearing defect observed in the presence of this particular mutation 73 (see **Table 1**).

**Table 1:** TABLE 1. Types of mitochondrial disease caused by mitochondrial encoded genes.

**Mitochondrial defects** - mitochondrial encoded (mtDNA) 60			
**Disease**	**Coding**	**Mutation**	**Reference**
Kearns Sayre Syndrome (**KSS**)	ND3, ND4, ND4L, ND5, COX3, ATP6, ATP8, tRNA^Leu^, tRNA^Ser^, tRNA^His^, tRNA^Arg^, tRNA^Gly^, tRNA^Lys^	Δ4977 (5 kb deletion)	13,14
Leigh Syndrome (**LS**)	ATP6	m.8993T>C	15
		m.9176T>G	16
	ND3	m.10158T>C	17,18,19
	ND4	m.11777C>A	20,15
	ND5	m.12706T>C	22
	ND6	m.14459G>A	23,24
		m.14487T>C	25,26
Leber Hereditary Optic Neuropathy (**LHON**)	ND4	m.11778G>A	27
	ND1	m.3460G>A	28,29
	ND6	m.14484T>C	30,31,32
Neurogenic Muscle Weakness, Ataxia and Retinitis Pigmentosa (**NARP**)	ATP6	m.8993T>G	33,34
Mitochondrial Encephalomyopathy, Lactid Acidosis and Stroke-like Episodes (**MELAS**)	ND1	m.3697C>A	35
	ND5	m.13513G>A	36
		m.13514A>G	37
	tRNA^Phe^	m.583G>A	38,39
	tRNA^Leu^ (UUR)	m.3243A>G	40
		m.3256C>T	41,42
		m.3271T>C	43,44,45
		m.3291T>C	46
	tRNA^Gln^	m.4332G>A	47
Myoclonic Epilepsy and Ragged Red Fiber Disease (**MERRF**)	tRNA^Lys^	m.8344A>G	48,49
		m.8356T>C	50,51,52
		m.8363G>A	53
Sensorineural Hearing Loss (**SNHL**)	tRNA^Ser^	m.7445A>G	54
		m.7511T>C	55
Deafness (**DEAF**)	12s rRNA	m.1494C>T	56
		m.1555A>G	57,58,59

Eye complications are also frequently found to be associated with mitochondrial dysfunction 74 and can be divided into primary and secondary. Primary afflictions are caused by genetic defects, whereas secondary afflictions are produce by hypertensive angiopathy of the retinal arteries, or diabetic retinopathy in mitochondrial diseases with diabetes 75. Mitochondrial optic neuropathies have been associated with mutations in mtDNA and in nuclear genes. The most frequent eye disorder due to mtDNA mutation is Leber´s hereditary optic neuropathy (LHON) 76. LHON usually affects young male adults and is characterised by mostly bilateral subacute or acute, painless, loss of central vision, with decreased colour vision^77^. There are three main mtDNA mutations that underly the majority of LHON cases and all of them are found in complex I genes: m.11778G > A in the ND4 gene, m.3460G > A in the ND1 gene, and m.14484T > C in the ND6 gene (see **Table 1**). In addition, these mutations are usually present in homoplasmy, indicating that probably other factors are involved in the development of the disorder. The molecular mechanisms underlying LHON are not yet fully understood. There have been some risks factors proposed, like specific mitochondrial haplogroups, smoking, alcohol consumption, and the use of some antibiotics. Differences in mitochondrial mass have been also postulated to play a role in the incomplete manifestation of the disease. LHON mutation carriers with no pathological phenotype have significantly higher mtDNA copy number in leukocytes than affected carriers. By comparing fibroblasts from unaffected and affected mutation carriers, along with controls, it was shown that unaffected carriers have increased mitochondrial transcripts, respiratory chain proteins and enzyme activities compared to controls and affected carriers. Therefore, increased mitochondrial mass may play a protective role in LHON and compensate for complex I dysfunction 78. In addition, males seem to be more affected because of the lack of protective effects from estrogen. Indeed, a study using cybrids carrying LHON mtDNA mutations showed that the addition of estradiol increased mitochondrial biogenesis and decreased ROS production by enhancing the activity of detoxifying enzymes like SOD2, leading to a decrease in apoptosis 79 (see **Table 1**).

The most common eye afflictions associated with nDNA mutations are autosomal dominant optic atrophy (ADOA), most frequently due to mutations in the Dynamin-like GTPase OPA1, and autosomal recessive optic atrophy (AROA), which has been mainly associated with mutations in the aconitate hydratase ACO2, or the uncharacterised transmembrane protein TMEM126A (OPA7) 76. ADOA is clinically characterised by bilaterally symmetric progressive deterioration of the central visual acuity. Approximately 60-70% of ADOA cases are caused by genetic alterations in OPA1, other genes implicated in this pathology are OPA2 80, OPA3 81, OPA4 82, OPA5 83, OPA8 84 and WFS1 85 (see **Table 1**). OPA1 is a protein with eight different isoforms, processed by the mitochondrial metallochaperones YME1L and OMA1 86,87,88. The best-known function of OPA1 is for inner mitochondrial fusion during mitochondrial dynamics. In addition, OPA1 is involved in the remodelling of cristae by tethering inter-cristae membranes and proper function of the protein is required for maintaining cristae structure 89. OPA1 mutations cause defective mitochondrial fusion and altered cristae structure, leading to direct effects on mitochondrial bioenergetics, including a decreased mitochondrial membrane potential and ATP synthesis and increased ROS production 90. Interestingly, deletion of YME1L in murine heart, which alters OPA1 processing and function in a tissue-specific way, causes dilated cardiomyopathy (**Figure 1**) 91.

AROA presents with progressive impairment of visual capacity. The defect could either be spontaneously recovered or may lead to bilateral and progressive blindness 77. Mutations in ACO2, affect the mitochondrial tricarboxylic acid cycle and therefore mitochondrial energy supply is depleted in patients 92. Although there have been several AROA patients with mutations in TMEM126A, the exact function of the protein and therefore the molecular mechanism underlying optic atrophy has yet to be determined 93,94,95.

### Heart 

Cardiac muscle has a high energetic demand, therefore cardiac complications are frequent among mitochondrial diseases. One of the most common cardiac afflictions present in these pathologies is cardiomyopathy, which is estimated to occur in 20-40% of children with mitochondrial disease 4,96,97. However, other symptoms like arrhythmia, conduction defects, pulmonary hypertension, dilated aortic root, pericardial effusion or coronary heart disease can also be developed as consequence of mitochondrial malfunction 5,98.

Mitochondrial cardiomyopathies are characterised by abnormal myocardial structure or function that results from genetic defects that impair the mitochondrial respiratory chain 6,98. Hypertrophic cardiomyopathy is the most common form, present in more than 50% of cases 7,96, but other forms, like dilated, restrictive, histiocytoid and left ventricular non-compactation cardiomyopathies can also be found among these patients 8,99.

As described before (see above), genetic defects affecting the integrity of respiratory chain complexes, mitochondrial translation, maintenance of mtDNA, lipid metabolism and other metabolic pathways inside mitochondria might lead to cardiac disease. Several important perturbations have been described in subunits or factors required for the proper assembly of respiratory chain complexes. In general, mutations in these proteins cause an impairment of respiration and ATP production, increased ROS production and finally, cellular stress derived from a bioenergetics impairment. To date, pathological mutations have been found in 26 structural subunits of complex I 9,100, that together with mutations in assembly factors represent around 30% of childhood mitochondrial diseases 11,101. Complex I defects can be present with isolated cardiomyopathy or together with multi-organic failure. Mutations in subunits of complex II or III have also been associated with different types of cardiomyopathy 12,102,103,104. Of special interest are defects of the cytochrome *c* oxidase, caused by mutations in assembly factors and in nuclear-encoded structural subunits. Mutations in the complex IV assembly factors COX10 and COX15 have been associated with hypertrophic cardiomyopathy 16,105. Both assembly factors are involved in the biosynthesis of heme A, the prosthetic group of the cytochrome *c* oxidase. Mutations in COX6B1, have been associated with cardiomyopathy and encephalopathy and showed decreased levels of the mature cytochrome *c* oxidase complex in patient-derived tissues and cells 12,106,107.

Although COX6B1 was thought to be a loosely interacting structural subunit of complex IV, studies have postulated Cox12 (yeast homolog of COX6B1) to be involved in the delivery of copper to Cox2, together with other metallochaperones like Sco1, Sco2 and Coa6 61,108. Indeed, mutations in human SCO2 have been mainly associated with cardioencephalopathy 62,109,110,111, whereas mutations in SCO1 have been associated with hepatic failure and encephalopathy 67,68,112,113, as well as cardiomyopathy 64,114. Both proteins contain a CXXXC that is able to coordinate copper. They bind to apo-COX2 and deliver two copper atoms to the Cu_A_ center. Both enzymes have different but cooperative functions and disruptions in their function impair the maturation of cytochrome *c* oxidase 70,115. In addition, the SCO1 and SCO2 proteins are involved in regulating cellular copper homeostasis 71,116. Recently, it has been shown that SCO1 keeps the copper transporter CTR1 in the plasma membrane, this function being essential for the development of adult myocardium in mice 72,117. Mutations in COA6 have been described in infants with hypertrophic cardiomyopathy and combined complex I and IV, or isolated complex IV deficiency in the heart 73,118,119. COA6 is required for cytochrome *c* oxidase assembly 74,120,121. It is involved in the insertion of copper into COX2 and it has been described to interact with SCO2 75,122 and SCO1 76,123 after the translocation of the COX2 C-terminal domain into the IMS by COX18 124. However, why disturbance of copper metabolism, or ultimately of the cytochrome *c* oxidase, specifically affects the heart remains unclear (see **Table 2**, **Figure 1**).

**Table 2:** TABLE 2. Mitochondrial defects caused by nuclear encoded genes.

**Mitochondrial defects** - nuclear encoded (nDNA) 60						
**OXPHOS** (**structural proteins** and assembly factors)						
Complex I		Complex II	Complex III	Complex IV		Complex V
**NDUFS1**	**NDUFS2**	**SDH-A**	**CYC1**	**COX4I1**	**COX4I2**	**ATP5E**
**NDUFS3**	**NDUFS4**	**SDH-B**	**UQCRC2**	**COX5A **	**COX6B1**	**ATP5A1**
**NDUFS6**	**NDUFS7**	**SDH-C**	**UQCRB**	**COX6A1**	**COX8A **	**ATP8A2**
**NDUFS8**	**NDUFB3**	**SDH-D**	**UQCRQ**	**COX7B**	COX15	ATPAF2
**NDUFB9**	**NDUFB10**	SDHAF1	BCS1L	SURF1	COX20	TMEM70
**NDUFB11**	**NDUFV1**	SDHF2	LYRM7	SCO1	COA3	
**NDUFA2**	**NDUFA9**		UQCC2	SCO2	COA5	
**NDUFA10**	**NDUFA11**		UQCC3	COX10	COA6	
**NDUFA12**	**NDUFA13**			COX14	COA7	
NDUFAF1	NDUFAF2			PET100	LRPPRC	
NDUFAF3	NDUFAF4			APOPT1	FASTKD2	
NDUFAF5	NDUFAF6				TACO1	
NUBPL	FOXRED					
ACAD9						
**mtDNA maintenance**						
POLG	POLG2	ANT1	MPV17	OPA1	MFN2	C10ORF2
FBXL4	FBXL4	RRM2B	SUCLA2	SUCLG1	TK2	TFAM
MGME1						
**Mitochondrial Import**						
DDP	DNAJC19					
**Mitochondrial Protein Synthesis **						
AARS2	CARS2	DARS2	EARS2	FARS2	GARS2	HARS2
IARS2	KARS	LARS	LARS2	NARS2	PARS2	RARS2
SARS2	TARS2	VARS2	YARS2	EFG1	TSFM	TUFM
GTPBP3	MTFMT	MTO1	TRMT5	TRMT10C	TRMU	GFM1
GFM2	C12orf65	RMND1	MRPL3	MRPS7	MRPL12	MRPS16
MRPS22	MRPL44	PUS1				
** Iron Homeostasis **						
FRDA	ABCB7	GLRX5	ISCU	BOLA3	NFU1	ISCA2
IBA57	LYRM4	LYRM7	FDXL1			
**Coenzyme Q10 biogenesis **						
COQ2	COQ4	COQ5	COQ6	COQ7	COQ9	APTX
PDSS1	PDSS2	CABC1				
** Mitochondrial quality control **						
SPG7	AFG3L2					
**Mitochondrial Integrity**						
DLP1	TAZ1	RMRP				
**Mitochondrial Metabolism **						
PDHA1	ETHE1	ATAD3				

Mutations in the mitochondrial translation machinery have also been associated with cardiomyopathy. Primary defects produce a decreased synthesis of mitochondrial polypeptides, but ultimately also impair mitochondrial bioenergetics and cause cellular stress. Mutations in the 16S mt-rRNA, and the m.1555A>G mutation in the 12S mt-rRNA, have been associated with hypertrophic and restrictive cardiomyopathy 78,125,126. Mutations in ribosomal proteins (MRPL3 and MRPL44) and the translation elongation factor (TSFM) can cause cardiomyopathy, together with multi-organic disease 79,127,128,129,130. Finally, defects in mitochondrial tRNAs can be linked to isolated cardiomyopathy or multi-organic disfunction 131,132,133 (see **Table 1** and **Table 2**).

Alterations of lipid metabolism inside mitochondria can also be a determinant for cardiac disease. Barth syndrome is an x-linked autosomal recessive disease, characterized by cardiomyopathy, skeletal myopathy, neutropenia, growth retardation, and 3-methylglutaconic acidurea 80,134,135,136 . This disorder is caused by mutations in the Tafazzin protein, TAZ1, a mitochondrial acyl-transferase involved in the biogenesis of cardiolipin (CL), a phospholipid almost exclusively found in the inner mitochondrial membrane 81,137. The adequate presence of CL is required for structural stability of many critical protein complexes in the mitochondrial membrane and it is therefore essential for many mitochondrial processes ranging from protein import, cristae morphology, function of the respiratory chain or cell stress signaling 82,136. Interestingly, oxidation of CL causes loss of interaction with cytochrome *c*, a pre-requisite for triggering apoptosis. Oxidized CL has been found to be involved in the opening of the mitochondrial permeability transition pore (MPTP). In addition, CL is exposed to the outer mitochondrial membrane during apoptosis, where it is used as a binding platform for pro-apoptotic factors. Therefore, CL homeostasis plays an important role in cardiomyocyte programmed death upon ischaemia or reperfusion (**Figure 1**) 83,136.

Mutations in another lipid related enzme, the acylglycerol kinase AGK, have been associated with hypertrophic cardiomyopathy, myopathy, cataracts, exercise intolerance and lactic acidosis (Sengers syndrome). AGK was recently described as a component of the carrier protein translocase of the inner membrane (TIM22) 84,138,139, meaning that a defective import of carrier proteins alters mitochondrial metabolism and may disturb the function of the heart.

### Neurological disorders

Similar to previously described organs and tissues and due to the high energy demands, neurological complications are commonly linked to mitochondrial disfunction. Indeed, some of the most known mitochondrial syndromes caused by abnormalities in the mtDNA present with drastic neurological symptoms: Kearns-Sayre syndrome (KSS), a multisystem disorder with progressive external ophthalmoplegia, pigmentary retinopathy, heart block and frequently other signs like ataxia, dementia or endocrine problems is associated with single deletions of mtDNA 140. MELAS (Mitochondrial Myopathy, Encephalopathy, Lactic Acidosis and Stroke-like Episodes) is caused in 80% of the cases by the m.3243A>G mutation in the tRNA^LEU(UUR)^ gene, although there have been other mutations described in protein coding genes 35,36,40,80,136,141. MERRF (Myoclonic Epilepsy and Ragged-Red Fiber), which usually also presents with cerebellar ataxia is mainly caused by mutations in the tRNA^lys^ gene (m8344A>G, m8356T>C, m.8363G>A), being the m.8344A>G the most frequent of them 49,51,53,142. The previously described defects affect the gene expression machinery of the mitochondrial genome and will generally affect mitochondrial protein synthesis, moreover an increased ROS production has been described in cybrids carrying the MELAS m.3243A>G mutation or the KSS associated common deletion Δ4977 89,143. In addition, there are mutations described in protein coding genes or in mitochondrial nuclear genes that would only affect individual complexes of the respiratory chain: NARP (Neuropathy, Ataxia and Retinitis Pigmentosa) has been mainly associated to the m.8933T>G/C mutation in the complex V subunit mt-ATP6 33. NARP patient derived cells were also found to have increased ROS production and decreased levels of ATP production 143. Leigh syndrome is a progressive neurometabolic disorder that usually presents with seizures, hypotonia, fatigue, nystagmus, poor reflexes, eating and swallowing difficulties, breathing problems, poor motor function, and ataxia. This unique mitochondrial disorder is found to be caused by both mutations in the mtDNA and the nDNA. Mutations in many different genes have been identified to be the origin of Leigh syndrome, including mtDNA subunits of the complex I, IV and V, mt-tRNAs, nuclear encoded subunits of complex I, IV and II, the pyruvate dehydrogenase complex, or some assembly factors of the cytochrome *c* oxidase (SURF1, SCO1, SCO2, COX10, COX15) or complex III (BSC1L) 144. Cells derived from patients with 3 different complex I mutations and Leigh syndrome exhibited increased ROS production 143,145 (see **Table 1** and **Table 2**, **Figure 1**).

As already mentioned, many more genes are being identified as the reason behind mitochondrial dysfunction. Defects in mtDNA maintenance may result into defective mtDNA replication and lead to quantitative loss of mtDNA (mtDNA depletion) or qualitative one (mtDNA deletion). Downstream perturbation of mitochondrial protein synthesis will final lead to a bioenergetics defect. MPV17 is a mitochondrial inner membrane protein involved in maintenance of mtDNA. It is believed to be involved in the import of deoxynucleotides into mitochondria. Pathogenic variants in MPV17 have been reported to cause hepatocerebral mtDNA depletion syndrome with liver failure, de velopment delay and other neurological manisfestations 146,147. In addition, infantile Navajo neuropathy (NNH), a neurohepatological disorder prevalently present among Navajo children in the southwestern of USA has been found to be caused by mutations in MPV17 148. A recent report analysing new pathological variants in MPV17 showed that most patients exhibited a single or combined respiratory chain complex activity decrease 149 (see **Table 2**).

Aminoacyl-tRNA synthetases (ARS), are a family of proteins encoded in the nucleus and present in either the cytosol or mitochondria that ensure the proper conjugation of an amino acid with its cognate tRNA molecules. All mt-ARS are synthesized in the cytosol, imported to mitochondria due to an N-terminal targeting sequence (presequence) which is cleaved upon translocation to the matrix. Pathogenic variants of mt-ARS will affect mitochondrial translation and have been implicated in human neurological disorders of the brain, spinal cord and motor neurons in addition to other symptoms. Some of the most typical presentations are leukoencephalopathy with involvement of the brainstem and spinal cord and high lactate due to mt-aspartyl-tRNA synthetase (DARS2) mutations 150, leukoencephalopathy with thalamus and brainstem involvement and high lactate, caused by mt-glutamyl- tRNA synthetase (EARS2 151). However, there are other mt-ARS mutations which may also produce white matter lesions. The similar symptoms shown by ARS mutations may imply a shared mechanism of disease, however such a mechanism has not been yet demonstrated. Among the possible molecular reasons are: a reduced aminoacylation activity, altered dimerization, mislocalization, gain of function though pathogenic interactions and loss of noncanonical function 152 (see **Table 2**).

## NEW STRATEGIES TO FIGHT MITOCHONDRIAL DERIVED STRESS

Nowadays there is no actual treatment for mitochondrial diseases. Nevertheless, in the last years a number of therapeutic strategies have been proposed, mainly in animal models. They can be classified into those acting on common pathways, and therefore applicable to different diseases, and those which aim to ameliorate a particular disorder (**Figure 1**) 153.

Those tissues or organs affected by decreased ATP production, and therefore impaired bioenergetics, can benefit from increased mitochondrial mass and activity. The transcriptional co-activator peroxisome proliferator activated receptor-1alpha (PGC1alpha) is the master regulator of mitochondrial biogenesis. It increases the activity of several transcription factors, like the nuclear respiratory factors (NR1 and NR2), thereby controlling the expression of OXPHOS related genes. In addition, PGC1alpha interacts with the peroxisomal proliferator activator receptors (PPARs), which regulate the expression of fatty acid oxidation genes 154. PGC1alpha is activated either by deacetylation by Sirt1, or phosphorylation by AMPK, both of which can be modulated pharmacologically 155.Under physiological conditions, PCG1alpha shows its highest expression levels in the heart, and mouse models lacking this protein have shown a normal cardiac function in unstimulated conditions. However, an impaired cardiac function was observed during certain stress conditions, like intense exercise or aortic constriction. Thus, the physiological role of PCG1alpha seems to be in fighting cellular stress 156.

Another possible strategy is to bypass the block in the respiratory chain from specific complex defects. In such a way, electrons would flow again and reduce ROS production. Concomitantly, unaffected complexes would pump protons across the inner membrane and increase ATP production. The yeast *Saccharomyces cerevisiae* NADH reductase (Ndi1), which transfers electrons from NADH to coenzyme *Q* (*CoQ*), has been used to bypass CI defects 157. In a similar approach, the alternative oxidase (AOX), which transfers electrons from *CoQ* to molecular oxygen in different organisms, has been used to bypass CIII and IV defects in cell culture 158 and to ameliorate to different extent respiratory defects in fly models 159,160. The enzyme has been successfully expressed in murine models 161, however correction of respiratory chain defects has not been shown yet *in vivo* in mammals.

As previously described (see above), the dynamin-like GTPAse OPA1 is required for proper mitochondrial shaping. Regulating fission and fusion helps fight mitochondrial malfunction. Increasing the expression of long isoforms of OPA1 improves respiration efficiency by enhancing supercomplex assembly and protects *in vivo* from many insults, such as ischemia/reperfusion, denervation/induced muscle atrophy, and OXPHOS deficiency 89,162,163.

In order to cope with increased oxidative damage generated in damaged mitochondria, different small molecules with antioxidant properties have been tested. Some examples, like Idebenone, lipoic acid, or Coenzyme Q_10 _, directly transfer electrons to the respiratory chain and bypass defective complexes. Others, like EPI-743 and RP103, enhance the biogenesis of glutathione, an important cellular antioxidant. KH176, can reduce altered cellular ROS levels and protect OXPHOS deficient cells against redox stress by targeting the Thioredoxin/Peroxiredoxin system 164. MTP-131 is a member of the Szeto‐Schiller (SS) peptide family and binds to CL. It increases OXPHOS capacity and improves the way mitochondria respond to metabolic changes. L-Arginine, a donor of nitric oxide, which thus regulates vascular tone, was shown to induce an improvement in aerobic capacity and muscle metabolism in models for mitochondrial disease 165.

Finally, genetic approaches can be used to correct mutations at a genomic level. Mitochondrially targeted restriction endonucleases have been used to shift heteroplasmy levels in cell lines with mutations in mtDNA and in heteroplasmic mice. Introduction of TALE and zinc finger nucleases (TALEN and ZFN) enabled the addition of specificity to the nucleases so that mutant DNA molecules could be selected for by directing unspecific restriction enzyme (FokI) to appropriate specific sequence assembling ZFN or TALE modules 166,167. However, this approach requires very large constructs that do not so easily fit into adeno-associated viruses (AVV) vectors. The Clustered Regularly Interspaced Short Palindromic Repeats (CRISPR) system is a bacterial immune system that has been modified for genome engineering. Due to the simplicity and adaptability of this technique, CRISPR has quickly displaced the previously established TALENs or ZFNs for genome engineering. CRISPR consists of two elements: a guide RNA (gRNA) and a non-specific CRISPR-associated endonuclease (Cas9). The gRNA is a short synthetic RNA composed of a "scaffold", necessary for Cas9 binding, and a 20 nt targeting sequence that is specific to the gene of interest 168. CRISPR was originally employed to knock-out target genes, but it has also been used to chromosomally modify or tag proteins, and to activate or repress target genes. However, the viability of this approach to target mitochondrial genes, mainly because of the requirement of a reliable nucleotide import system into mitochondria is not yet clear 169.

## CONCLUDING REMARKS

Mitochondrial diseases show very complex and various clinical presentations. Because of the dual genetic origin of mitochondrial proteins, the number of genes susceptible of causing a mitochondrial pathology is large. Thus, the diagnostic process of mitochondrial diseases is usually complicated and very long, and in many cases although there is a clear suspicion of a mitochondrial defect, the final defect behind the phenotype remains undercover 170.

Despite the heterogeneity of the diseases and the genetic defects the final kind of cellular stresses are similar. In the most cases, there is a bioenergetic defect or an increased production of ROS. It remains unclear why although many genetic defects are present in the whole body, only certain tissues are affected. There may be different mechanisms to cope with mitochondrial stress, which may be tissue specific 73. Indeed, disease models of mtDNA replication machinery failure have been linked to imbalance of the cellular dNTPs pool and consequently to increased glutathione biogenesis through *de novo* serine biogenesis. This metabolic switch was proposed to be a specific and rapid response to cellular stress/mtDNA damage in skeletal muscle and heart 171. Together with this pathway, transcriptional response and mitochondrial unfolded protein response constitute the integrated mitochondrial stress response (ISRmt), which is controlled by the metabolic signalling regulator mTORC1 in muscle. However, long-term activation of cellular stress responses may be detrimental since chronic upregulation of anabolism contributes to mitochondrial myopathy pathogenesis 172. In addition, there is a certain multifactorial component in mitochondrial diseases. In some cases, ancient mt-rRNAs mutations rendered no adverse phenotype unless some environmental factors were used 72. Therefore, the different incorporation and tolerance of tissues and organs for different xenobiotics may be different. Population variation plays also an important role in enhancing/ diminishing mitochondrial-related phenotypes, like population polymorphisms in mt-rRNAs and side effects derived from antibiotic treatment 173. Nevertheless, a deeper investigation will be required to understand tissue specificity of mitochondrial diseases.

There have been several approaches proposed to treat mitochondrial diseases, however their application to the clinics is still a challenge 153. Nevertheless, the discovery of new genome editing tools and the development of stem cell technologies will provide open new avenues of possibilities for the treatment of mitochondrial diseases.

## Abbreviations

ADOA: autosomal dominant optic atrophy
AROA: autosomal recessive optic atrophy
ARS: aminoacyl-tRNA synthetase
CL: cardiolipin
CRISPR: clustered regularly interspaced short palindromic repeats
LHON: Leber’s hereditary optic neuropathy
mt: mitochondrial
OXPHOS: oxidative phosphorylation
ROS: reactive oxygen species
